# The Value of a Rapid Test of Human Regulatory T Cell Function Needs to be Revised

**DOI:** 10.3389/fimmu.2019.00150

**Published:** 2019-02-05

**Authors:** Desiree J. Wendering, Leila Amini, Stephan Schlickeiser, Petra Reinke, Hans-Dieter Volk, Michael Schmueck-Henneresse

**Affiliations:** ^1^Institute for Medical Immunology, Charité–Universitätsmedizin Berlin, Berlin, Germany; ^2^Berlin Center for Advanced Therapies (BeCAT), Charité–Universitätsmedizin Berlin, Berlin, Germany; ^3^Berlin Institute of Health (BIH) Centrum for Regenerative Therapies (B-CRT), Charité–Universitätsmedizin Berlin, Berlin, Germany

**Keywords:** regulatory T cell functional assay, αCD3/28-coated microbeads, competitive CD3/CD28 binding, nullified Treg-mediated suppression, correlation between T cell-to-αCD3/CD28-coated microbead ratio and activation marker frequency on responder T cells

## Abstract

CD4^+^CD25^+^FoxP3^+^ human regulatory T_CELLS_ (T_REG_) are promising candidates for reshaping undesired immunity/inflammation by adoptive cell transfer, yet their application is strongly dependent on robust assays testing their functionality. Several studies along with first clinical data indicate T_REG_ to be auspicious to use for future cell therapies, e.g., to induce tolerance after solid organ transplantation. To this end, T_REG_ suppressive capacity has to be thoroughly evaluated prior to any therapeutic application. A 7 h-protocol for the assessment of T_REG_ function by suppression of the early activation markers CD154 and CD69 on CD4^+^CD25^−^ responder T_CELLS_ (T_RESP_) upon polyclonal stimulation via αCD3/28-coated activating microbeads has previously been published. Even though this assay has since been applied by various groups, the protocol comes with a critical pitfall, which is yet not corrected by the journal of its original publication. Our results demonstrate that the observed decrease in activation marker frequency on T_RESP_ is due to competition for αCD3/28-coated microbeads as opposed to a T_REG_-attributable effect and therefore the protocol cannot further be used as a diagnostic test to assess suppressive T_REG_ function.

## Introduction

Regulatory T_CELLS_ (T_REG_) are key players in maintaining immune homeostasis, resolution of inflammation, and self (1). Exploiting those characteristics, T_REG_ have gained plenty of attention as promising candidates in immunotherapeutic applications for the prevention or reshaping of undesired immune responses such as in autoimmune diseases, chronic inflammation, and allograft rejections. Data from clinical trials identify T_REG_ as an encouraging cell type for use in cellular therapy (2). By the same token, a robust protocol to assess T_REG_ function is of utmost importance to ensure their suppressive function prior to adoptive cell-therapeutic clinical trials, as well as for application in basic research. So far, for assessing T_REG_ functionality, evaluating the suppressive capacity of T_REG_ to inhibit the proliferation of responder T_CELL_ (T_RESP_) after a 4-day co-cultivation period has been the gold-standard protocol since a decade (3, 4). Recently, Canavan et al. (5) and Ruitenberg et al. (6) described a rapid 7 h assay for the evaluation of T_REG_ functionality by assessing their suppressive capacity using upregulation of the early T_CELL_ activation makers CD154 (CD40L) and CD69 on conventional CD4^+^CD25^−^ responder T_CELLS_ (T_RESP_) upon CD3/28 engagement. CD3/28 stimulation is mediated by microbeads coupled with αCD3 and αCD28 antibodies. According to these studies, T_REG_ alleviate CD154 and CD69 expression on T_RESP_ in a dose-dependent manner. Even though this assay has since been frequently applied and cited more than 80 times (7, 8, 10), we observed that the protocol comes with a critical pitfall: T_RESP_ and T_REG_ both express the signaling molecule CD3 and T_CELL_ co-stimulatory receptor CD28 on the plasma membrane, potentially competing for binding αCD3/28 T_CELL_ activating microbeads applied in the rapid 7 h assay. We investigated whether the observed decreased frequencies of activated T_RESP_ can be claimed to be a T_REG_-attributable effect or if it is rather a result of competition for αCD3/28-coated activating microbeads. We thus explored whether different ratios of αCD3/28 T_CELL_ activation microbeads-to-T_CELLS_ impact the outcome of this functional T_REG_ assay.

## Materials and Methods

### Study Design

The aim of this study was to investigate the influence of αCD3/CD28-coated activating microbeads on the expression of early activation markers CD69 and CD154, used for predicting T_REG_ functionality in basic and translational research. We compared the expression of CD69 and CD154 of T_RESP_ in T_REG_ co-cultures, which were either activated via αCD3/CD28-coated microbeads adjusted to T_RESP_ only or to the total cell number present in one well (T_RESP_ + T_REG_). To verify the integrity of the T_REG_ used in this study, as well as to demonstrate the T_REG_-mediated suppressive function in a bead-uncompetitive setting, T_RESP_ proliferation suppression experiments were performed.

### Cell Isolation

Peripheral blood mononuclear cells from healthy donors were purified using Ficoll-Paque separation (Biochrom). CD4^+^ cells were enriched by magnetic-activated cell sorting (Miltenyi) according to manufacturer's instructions (purity>90%). For fluorescence-activated cell sorting (FACS Aria II, BD) of CD4^+^CD25^high^CD127^low^ T_REG_ and CD4^+^CD25^−^ T_RESP_, cells were stained with CD4 (SK3, Biolegend), CD25 (2A3, BD), and CD127 (R34.34, Beckman Coulter). Post-FACSort analysis by flow cytometry yielded CD25^+^FoxP3^+^ T_CELL_ purity of >95%.

### 7 h Diagnostic Test for T_**REG**_ Function and αCD3/28 Microbead Titration

Assays were performed as described by Canavan et al. (5). Briefly, CFSE-labeled T_RESP_ were co-cultured with autologous T_REG_ at T_RESP_/T_REG_ ratios ranging from 1:1 to 32:1. In two parallel setups, cells were either stimulated with αCD3/28-coated microbeads (Dynabeads® Human T-Activator CD3/CD28, Thermo Fisher Scientific) at a bead/cell ratio of 0.2 adjusted to the T_RESP_ cell number per well (5, 6) or adapting the ratio of 0.2 to the total cell number per well including T_REG_. Stimulated and unstimulated T_RESP_ without T_REG_ were included as controls. For the microbead titration, T_RESP_ were cultured alone at bead/T_RESP_ ratios ranging from 0.1 to 0.4 (mimicking the presence of T_REG_). αCD154 (24–31) was added at start of incubation. Cells were incubated at 37°C for 7 h. All cell cultures were performed in *X-Vivo*-15 medium supplemented with 10% FCS (Lonza & Biochrom) and 100 IU/ml Penicillin/Streptomycin. After harvesting, cells were stained with CD3 (OKT3), CD4 (SK3), CD137 (4B4-4), and CD69 (FN50), all Biolegend. Dead cells were excluded (LIFE/DEAD^TM^ Fixable Blue Dead Cell Stain Kit, Thermo Fisher Scientific).

#### Proliferation Suppression Assay

CFSE-labeled T_RESP_ were cultured alone or with autologous T_REGS_ at T_RESP_/T_REG_ ratios ranging from 1:1 to 16:1. The cells were stimulated with αCD3/28-coated microbeads (T_REG_ Suppression Inspector, Miltenyi) at a cell/bead ratio of 1:1 and 1:2 adjusted to the total cell number per well and incubated at 37°C for 96 h. Thereafter, cells were stained with CD3 (OKT3), CD4 (SK3), all Biolegend. Dead cells were excluded (Thermo Fisher Scientific). Proliferation was assessed by CFSE dilution and percentage suppression of proliferation was calculated by relating the percentage of proliferating T_RESP_ in the presence and absence of T_REG_, respectively.

#### Flow Cytometry Analysis

Data were acquired on a LSR-II Fortessa flow cytometer (BD) and analyzed using FlowJo V10 (TreeStar).

#### Statistics

Analysis was performed with GraphPad Prism software (version 6, GraphPad, La Jolla, CA) and R (version 3.4.1) (9). We have tested for significant interaction, i.e., non-parallel response profiles of the two bead adjustment methods to the different T_RESP_:T_REG_ ratios, using a non-parametric rank-based ANOVA-type statistic [as implemented in the *nparLD* package (11)] in a two-way factorial repeated measures design. For bead titration experiments, non-parametric two-tailed Wilcoxon matched-pairs signed rank tests were used to determine significance in pairwise comparison. Data indicate means ± SEMs in all bar graphs. *P* < 0.05 was considered significant.

## Results

### T_**CELL**_ Early Activation Marker Expression Is Dependent of TCR Engagement

**Figure 1:**
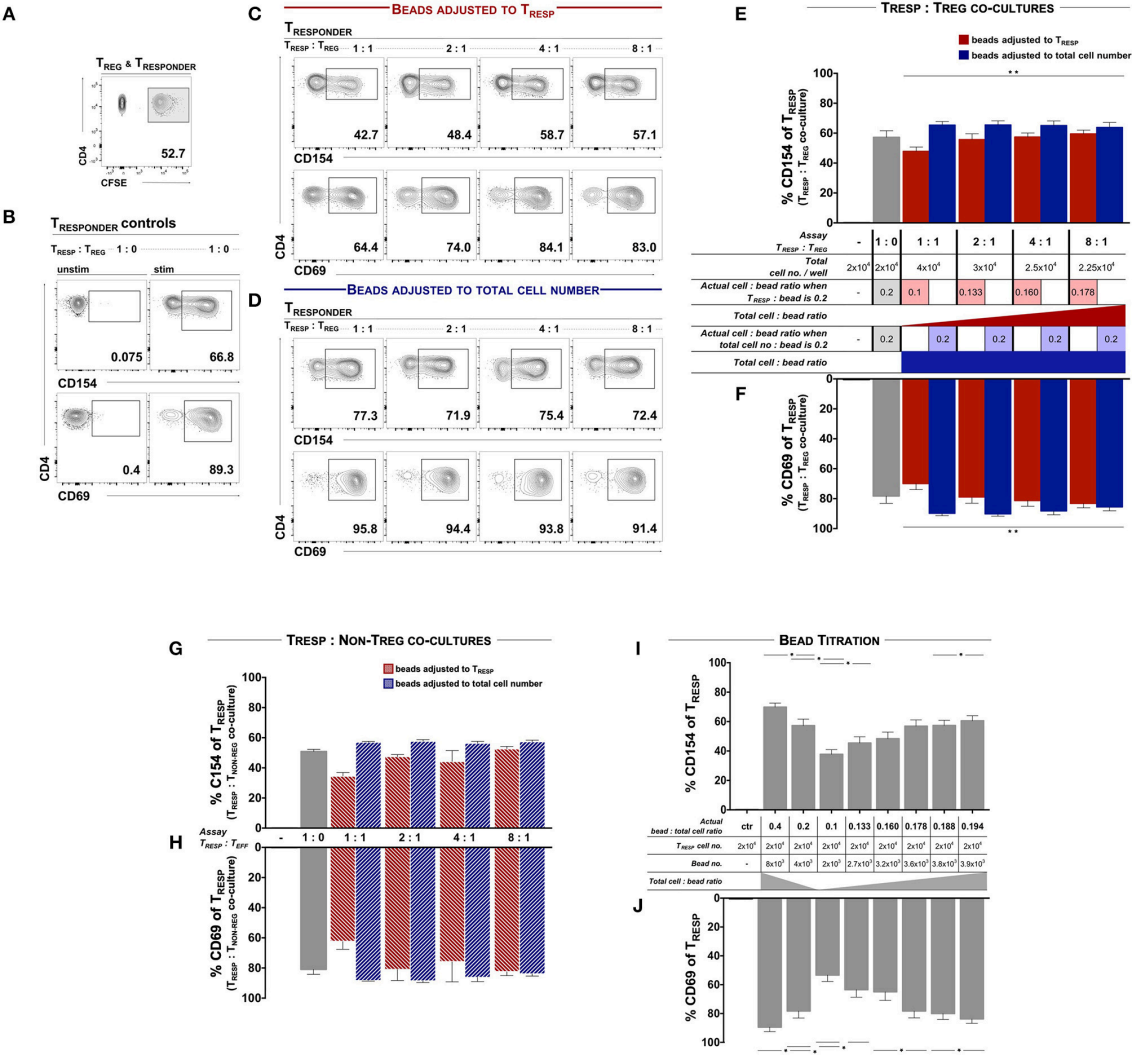
T_CELL_ early activation marker expression is dependent of TCR engagement and cannot be used for T_REG_ functional evaluation. FACSorted CD4^+^CD25^−^ T_RESP_ with and without autologous T_REG_ co-culture were stimulated with anti-CD3/CD28-coated microbeads and analyzed for early activation marker expression. **(A)** For precise T_RESP_/T_REG_ discrimination, T_RESP_ were labeled with CFDA-SE (CFSE). **(B)** Representative plots of CD154 and CD69 expression on unstimulated and stimulated T_RESP_ cultured without T_REG_. **(C)** Representative plots of CD154 and CD69 expression of T_RESP_ co-cultured with T_REG_ at different T_RESP_:T_REG_ ratios and stimulated with anti-CD3/CD28-coated microbeads adjusted to T_RESP_. **(D)** Representative plots of CD154 and CD69 expression of T_RESP_ co-cultured with T_REG_ at different T_RESP_:T_REG_ ratios and stimulated with anti-CD3/CD28-coated microbeads adjusted to total cell number. **(E,F)** Quantified data from **(C,D)**, respectively. CD154 and CD69 of CFSE^+^T_RESP_ co-cultured with FACSorted T_REG_ at different T_RESP_:T_REG_ ratios and stimulated with anti-CD3/CD28-coated microbeads adjusted to T_RESP_ (red columns) and to total cell numbers (blue columns). For clarification, the table summarizes the experimental setups. *n* = 7. Non-parametric rank-based ANOVA-type statistic ^**^p < 0.001 (CD154: *p* = 1.90E-06, CD69: *p* = 5.527256E-16). **(G)** Expression of CD154 and **(H)** expression of CD69 of CFSE^+^T_RESP_ co-cultured with FACSorted T_NON−TREG_ at different T_RESP_:T_REG_ ratios and stimulated with anti-CD3/CD28-coated microbeads adjusted to T_RESP_ (red columns) and to total cell numbers (blue columns). *n* = 3. **(I)** Expression of CD154 and **(J)** expression of CD69 of CFSE^+^T_RESP_ after different anti-CD3/CD28-coated microbead:T_RESP_ ratio stimulation. For clarification, the table summarizes the experimental setups. *n* = 7. ^*^p < 0.05, Wilcoxon matched-pairs signed rank test. T_RESP_:T_REG_/T_NON−TREG_ co-cultures **(E,F)** and corresponding bead titration **(I,J)** experiments were performed simultaneously using the same donor cells. Median data of independent experiments are shown and error bars represent SEM.

We first examined T_REG_ functionality according to the protocols published by Canavan et al. (5) and Ruitenberg et al. (6), whereby *ex vivo* FACSorted and CFSE-labeled T_RESP_ were co-cultured in the presence and absence of autologous T_REG_ and stimulated with αCD3/28-coated activating microbeads at a ratio of 0.2 microbeads per T_RESP_ (Figure 1A). After 7 h, the mean frequency of CD154^+^ and CD69^+^ T_CELLS_ of unstimulated T_RESP_ was 0.14 and 0.45%, respectively and 57.25 and 78.26% on CD3/28-stimulated T_RESP_, respectively (Figure 1B). When T_RESP_ were stimulated in the presence of T_REG_ at ratio 1:1, the mean frequency of CD154^+^ and CD69^+^ T_CELLS_ decreased to 47.77 and 69.86%, respectively. With increasing T_RESP_/T_REG_ ratios both, CD154 and CD69 expression, increased in a linear fashion (Figure 1C, quantified in E, F, red columns). We next determined whether the total T_CELL_/bead ratio influences T_REG_-induced activation marker suppression. Accordingly, we adjusted the bead numbers to the total cell numbers, including T_REG_, thereby eluding the bead competition in contrast to Canavan et al. (5) and Ruitenberg et al. (6). In that case, T_RESP_ activation in the presence of T_REG_ equaled control T_RESP_ cultures without T_REG_ (Figure 1D, quantified in E, F, blue bars), indicating that indeed T_RESP_ and T_REG_ compete for CD3/28-binding microbeads. Serving as a negative control, we co-cultured T_RESP_ with CD4^+^CD25^−^ non-T_REG_/effector T_CELLS_ in place of T_REG_. When the bead number was adjusted to T_RESP_ only we observed similar reductions of CD154 and CD69 expression (Figures 1G,H, red bars) as when T_RESP_ were co-cultured with T_REG_ (Figures 1E,F, red bars). Correspondingly, when adjusting the bead number to the total cell number (Figures 1E,H, blue bars), the expression of CD154 and CD69 is similar to the conditions with T_RESP_ only (Figures 1E–H, gray bars). To mimic the competition for the activating microbead stimuli, we stimulated T_RESP_ with different amounts of αCD3/28-coated microbeads in the absence of T_REG_. We set the actual bead/T_CELL_ ratio according to the published T_RESP_/T_REG_ co-culture approach, in which the activation bead/T_RESP_ ratio is adjusted to T_RESP_ only, i.e., calculated the actual bead/T_CELL_ ratio in each setting. CD154 and CD69 expression decreased in a dose-dependent manner with highest expression levels at a bead/T_RESP_ ratio of 0.4 (69.83 and 89.47%, respectively) and lowest at a ratio of 0.1 (37.80 and 53.33%, respectively). The T_RESP_ activation pattern with the different bead ratios ranging from 0.1 to 0.194 indicate a strong bead/T_RESP_ ratio dependency (Figures 1I,J).

### T_**REG**_ Demonstrate a Dose-Dependent T_**RESP**_ Proliferation Suppression in a Bead-Uncompetitive Setting

**Figure 2:**
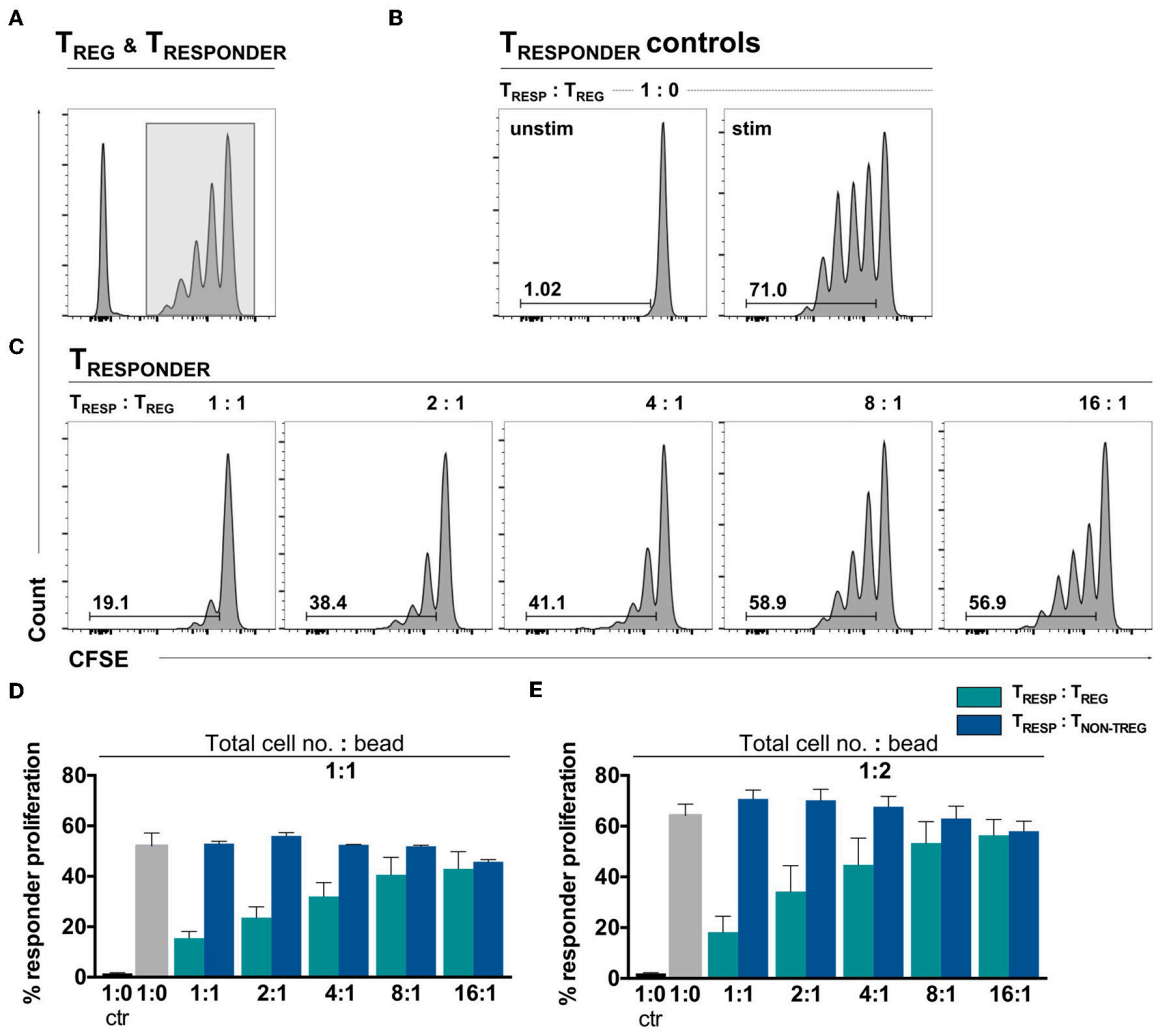
*Ex vivo* isolated T_REG_ demonstrate a dose-dependent T_RESP_ proliferation suppression in a bead-uncompetitive setting. FACSorted CFSE^+^CD4^+^CD25^−^ T_RESP_ were co-cultured with autologous FACSorted T_REG_ and stimulated with anti-CD3/CD28-coated microbeads for 96 h. T_RESP_ proliferation was analyzed by CFSE dilution. Representative plots depicting **(A)** CFSE-labeling strategy to accurately analyze T_RESP_ proliferation; **(B)** proliferation of unstimulated and stimulated CFSE^+^T_RESP_ cultured without T_REG_ and **(C)** CFSE^+^T_RESP_ proliferation after co-culture with different T_REG_ ratios. **(D)** Percentage of T_RESP_ proliferation after co-culture with decreasing T_RESP_:T_REG_ ratios (green bars) and T_RESP_:T_NON_-_TREG_ ratios (blue bars) stimulated with a total cell number:bead ratio of 1:1. *n* = 7 T_RESP_:T_REG_ co-cultures, *n* = 3 T_RESP_:T_NON−TREG_ co-cultures. **(E)** Percentage of T_RESP_ proliferation after co-culture with decreasing T_RESP_:T_REG_ ratios (green bars) and T_RESP_:T_NON_-_TREG_ ratios (blue bars) stimulated with a total cell number:bead ratio of 1:2. *n* = 3. Median data of independent experiments are shown and error bars represent SEM.

To confirm T_REG_ functionality in an environment where the number of αCD3/28-activation microbeads is adjusted to the total cell number, the gold-standard T_RESP_ proliferation suppression assay was performed. The proliferation assay was conducted with T_CELLS_ of the same donors in parallel to the experiments shown in Figure 1. Following activation, T_RESP_ proliferation alone yielded 52.03% and dose-dependently decreased in the presence of T_REG_ to 15.51% at a T_RESP_/T_REG_ ratio of 1:1 (Figures 2A–C, quantified in Figure 2D, green bars). Thus, we conclude that the T_REG_ employed in this study are able to suppress T_RESP_ proliferation in a standardized bead-competitive setting. To ascertain the reduction of proliferation to be T_REG_-mediated, we have added non-T_REG_/effector T_CELLS_ instead of T_REG_ to T_RESP_ and observed no decrease in T_RESP_ proliferation, indicating the suppression of T_RESP_ proliferation to be a T_REG_-attributable effect (Figure 2D, blue bars). Even when T_CELLS_ are stimulated with twice the number of activating αCD3/CD28 microbeads, the T_REG_-specific impact in suppressing T_RESP_ proliferation can be seen (Figure 2E).

## Discussion

In conclusion, when adjusting the αCD3/28-bead numbers to only T_RESP_ in co-cultures of T_RESP_ and T_REG_, activation marker expression was comparable to approaches where T_RESP_ were cultured alone at same bead/total cell ratio present in the T_RESP_/T_REG_ co-culture. When normalizing αCD3/28-bead competition by adjusting the bead number to total cell numbers, T_REG_-mediated suppression of activation marker upregulation is nullified. Even more strikingly, when titrating non-T_REG_/effector T_CELLS_ to T_RESP_ and adjusting the αCD3/28-bead numbers to T_RESP_ only, we observe the same decrease in activation marker expression as in T_RESP_:T_REG_ co-cultures. We thereby demonstrate that the suppression of activation marker expression on T_RESP_ observed in co-cultures with T_REG_ are due to competitive T_CELL_ receptor and CD28 engagement limited by αCD3/28 microbead availability rather than by suppressive activity of T_REG_ (Supplementary Figure 1). There is a pressing demand for a fast assay to evaluate T_REG_ functionality, especially in the light of upcoming clinical trials needing a robust diagnostic test to assess the suppressive function as a release criterion for their T_REG_ cell products. Nonetheless, the T_RESP_ proliferation suppression analysis should still be considered as the gold-standard T_REG_ functional assay as it is performed by adjusting the activation bead to T_CELL_ ratios in experimental setups with decreasing T_REG_ cell numbers (to assess T_REG_ dose-dependent suppression). Since we firmly believe that activation bead to T_CELL_ receptor competition should be kept constant throughout all conditions within a T_REG_ functional assay, we claim that the rapid assessment for human T_REG_ function proposed by Canavan et al. (5) and Ruitenberg et al. (6) does not result in reliable evidence of functional suppression since the putative T_REG_-mediated suppression of T_RESP_ activation is to be ascribed to competitive T_CELL_ receptor and CD28 engagement. Hence, we suggest that the previously published protocol is unsuitable as a diagnostic test to assess suppressive T_REG_ function.

## Ethics Statement

The Charité Ethics Committee (IRB) approved the study protocol and all blood donors provided written informed consent.

## Author Contributions

DW designed the research, performed experiments, analyzed and interpreted the data, and wrote the manuscript. LA performed experiments and revised the manuscript. SS performed statistical analyses. PR revised the manuscript. H-DV interpreted the data and revised the manuscript. MS-H led the project, designed the research, analyzed and interpreted the data, and wrote the manuscript.

### Conflict of Interest Statement

The authors declare that the research was conducted in the absence of any commercial or financial relationships that could be construed as a potential conflict of interest.
