# Supplementary material for: The Value of a Rapid Test of Human Regulatory T Cell Function Needs to be Revised
Source: Front Immunol. 2019 Feb 5;10:150. doi: 10.3389/fimmu.2019.00150 (PMC6370705; doi:10.3389/fimmu.2019.00150)
Supplement: Supplementary file 1 [file Image_1.pdf]

Supplementary Material

The value of a rapid test of human regulatory T cell function needs to be revised

Desiree J. Wendering, Leila Amini, Stephan Schlickeiser Petra Reinke, Hans-Dieter Volk, Michael Schmueck-Henneresse[1*]

*** Correspondence:** Corresponding Author: michael.schmueck-henneresse@charite.de

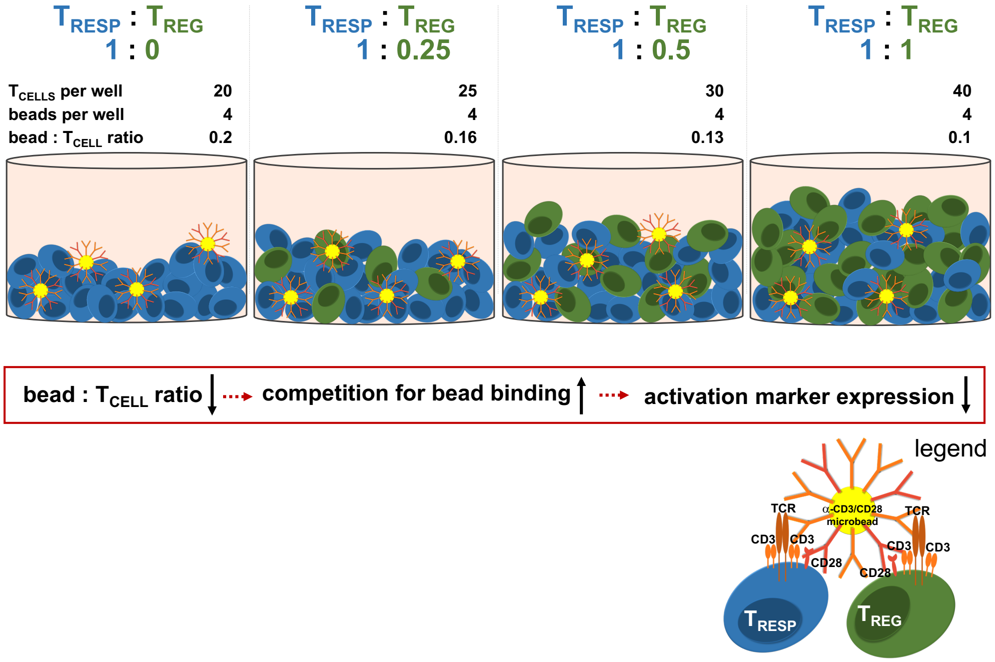

Supplementary Figure 1. Schematic diagram of T_{CELL} / αCD3/28-coated activating microbead competition in dependence of microbead/T_{CELL}

Since T_{RESP} cell numbers are kept constant and T_{REG} are titrated to T_{RESP} in a functional T_{RESP}/T_{REG} co-culture assay, also activating microbead numbers are constant when adjusted to T_{RESP} only – in this case 4 beads per 20 T_{RESP} and therefore a microbead/T_{CELL} ratio of 0.2. When the same amount of T_{REG} is added to T_{RESP} in a 1:1 co-culture setting, the actual microbead /T_{CELL} ratio is halved, i.e. decreased to 0.1. By decreasing the T_{REG} number to demonstrate a T_{REG}-specific effect, the total T_{CELL} numbers per well decrease and consequently lead to an increase in microbead /T_{CELL} ratio. Both, T_{RESP} and T_{REG} express the signaling molecule CD3 and T_{CELL} co-stimulatory receptor CD28 on their cell surface. As therefore both T_{CELL} populations compete for binding αCD3/CD28-coated activating microbeads, highest microbead availability per T_{CELL} would predict the greatest T_{CELL} activation and hence the lowest T_{CELL} activation when bead availability is lowest.
